# Cytoplasmic region of beta-dystroglycan is essential for postsynaptic maturation and neuromuscular function in mice

**DOI:** 10.1073/pnas.2600931123

**Published:** 2026-06-03

**Authors:** Jeffrey M. Hord, Rolf Turk, Hajime Kusano, Erik P. Rader, Sarah Burns, Zeita Gastel, Sally J. Prouty, Liping Yu, Steven J. Burden, Kevin P. Campbell

**Affiliations:** ^a^ Senator Paul D. Wellstone Muscular Dystrophy Specialized Research Center, Department of Molecular Physiology and Biophysics, University of Iowa Roy J. and Lucille A. Carver College of Medicine, Iowa City, IA 52242; ^b^Department of Physiology, College of Graduate Studies, Midwestern University, Glendale, AZ 85308; ^c^ Department of Molecular Physiology and Biophysics, University of Iowa Roy J. and Lucille A. Carver College of Medicine, Iowa City, IA 52242; ^d^ Department of Neurology, University of Iowa Roy J. and Lucille A. Carver College of Medicine, Iowa City, IA 52242; ^e^ Department of Orthopaedics, West Virginia University School of Medicine, West Virginia University, Morgantown, WV 26506; ^f^ Department of Biochemistry and Molecular Biology, University of Iowa, Iowa City, IA 52242; ^g^ Nuclear Magnetic Resonance Core Facility, Carver College of Medicine, Department of Biochemistry and Molecular Biology, University of Iowa, Iowa City, IA 52242; ^h^ Department of Neurology, Massachusetts General Hospital, Boston, MA 02114

**Keywords:** dystroglycan, neuromuscular junction, skeletal muscle, dystroglycanopathy, muscular dystrophy

## Abstract

The dystrophin–glycoprotein complex (DGC) provides structural integrity to the sarcolemma, and disruption of the DGC leads to muscular dystrophy. A core member of the DGC is dystroglycan (DG), which binds to extracellular ligands via α-DG and intracellular cytoskeleton via β-DG. Mutations in *DAG1* or genes involved in the posttranslational processing of DG lead to a subset of neuromuscular diseases referred to as dystroglycanopathies. The importance of the α-DG extracellular interactions is well established; however, little is known about the significance of the β-DG intracellular interactions. Here, we investigate the importance of intracellular β-DG in neuromuscular health. Using a mouse that lacks a large intracellular region of β-DG (residues 777 to 893), we show that the deletion of cytoplasmic β-DG leads to skeletal muscle pathology accompanied by postsynaptic disruption. Our data show that within the specialized neuromuscular junction (NMJ), cytoplasmic β-DG is necessary for the localization of utrophin and rapsyn, and clustering of acetylcholine receptors. Moreover, we provide evidence that the postsynaptic abnormalities contribute to neuromuscular dysfunction in mice lacking the cytoplasmic region of β-DG. Further, using a mouse model that only lacks the C-terminal tail (residues 879 to 893) of β-DG, we demonstrate that skeletal muscle and NMJ health rely on β-DG residues 777 to 878. Together, our mouse models suggest that deletion of the cytodomain of β-DG surprisingly results in very severe neuromuscular pathophysiology in mice. Our results identify β-DG as a critical player in shaping and maintaining neuromuscular synapse architecture in vivo, thus further defining the molecular mechanisms underlying neuromuscular health.

## Significance

Neuromuscular health depends on how well motor neurons and skeletal muscle fibers work together to produce muscle strength and movement, and to support overall physical health. The neuromuscular junction, a specialized connection that links motor neurons to muscle fibers, enables excitatory signals from the neurons to trigger muscle contractions. Our study shows that a protein called dystroglycan plays an essential role in maintaining the structure of this connection. When only intracellular dystroglycan is removed, mice develop severe neuromuscular disease. The present findings highlight the physiological significance of intracellular dystroglycan in the synaptic membrane of skeletal muscle. Overall, our research provides insights into the contribution of postsynaptic disruption to neuromuscular disease.

The dystrophin–glycoprotein complex (DGC) is a multimeric complex that provides stability and integrity to the sarcolemma and protection from mechanical/contractile stress through its linkage from the extracellular matrix (ECM) to the cytoskeleton (1–5). The DGC includes cytosolic dystrophin and dystrobrevin, transmembrane sarcospan, along with the sarcoglycans (α-, β-, γ-, δ-SG) and dystroglycan (α- and β-DG) that span intracellularly to extracellularly (*SI Appendix*, Fig. S1*A*) (6, 7). Two recent structural analyses of the DGC discovered that sarcospan and sarcoglycans flank either side of DG (8, 9), likely to help stabilize DG. The structural insights demonstrate that DG is the central pillar and essential for providing stability and integrity to the membrane. The *DAG1* gene encodes DG, which consists of α-DG and β-DG subunits. DG creates a vital connection between the surrounding ECM and the intracellular cytoskeleton (2, 3). *O*-mannosyl modification of the mucin-like domain of α-DG culminates in a heteropolysaccharide known as matriglycan that is responsible for high affinity binding to ECM proteins such as laminin, agrin, and perlecan. Intracellularly, β-DG interacts with dystrophin, utrophin, and rapsyn, thus establishing a connection to the submembrane cytoskeleton (*SI Appendix*, Fig. S1 *A* and *B*) (8–13).

**Fig. 1.**
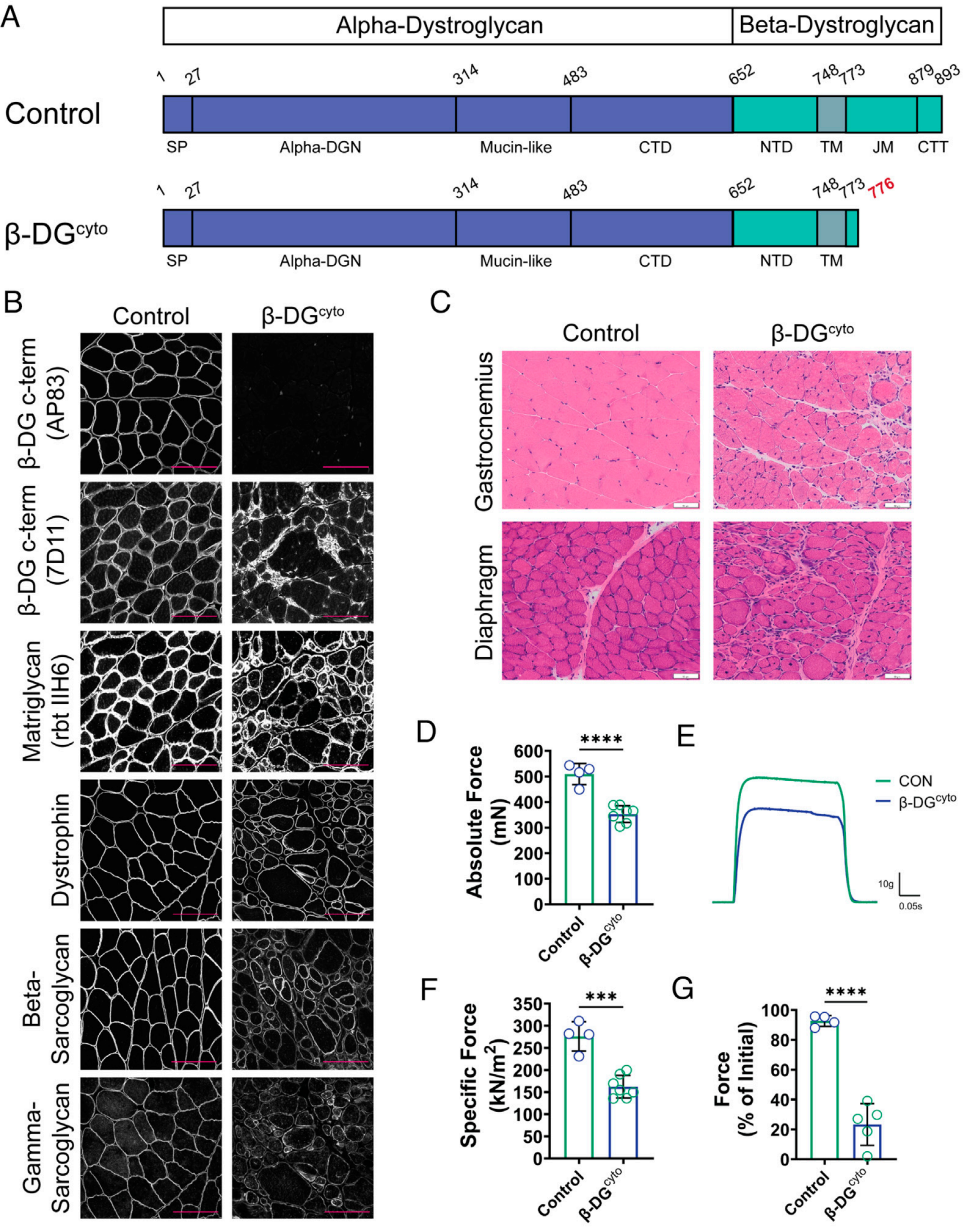
Deletion of the cytoplasmic region of β-DG leads to skeletal muscle pathology in mice. (*A*) Depiction of the multiple domains within DG. SP, signal peptide; Alpha-DGN, N-terminal domain of α-DG; CTD, C-terminal domain of α-DG; NTD, N-terminal domain of β-DG; TM, transmembrane domain of β-DG; JM, juxtamembrane domain of β-DG; CTT, carboxy-terminus tail of β-DG. Illustrative comparison of control and β-DG^cyto^ alleles demonstrate the lack of the final 117 residues of β-DG (ending at residue 776, as indicated by red font) in the β-DG^cyto^ mice. (*B*) Immunofluorescence of gastrocnemius muscle cryosections probing for the carboxy-tail of β-DG with the rabbit-derived antibody AP83 (*top* *row*) and the mouse-derived antibody 7D11 (*second row*); matriglycan, the *O*-mannosyl modification on α-DG responsible for binding to ECM ligands, with the rabbit antibody IIH6 (*third row*); dystrophin (*fourth row*); β-Sarcoglycan (*fifth row*); and γ-Sarcoglycan (*bottom* *row*). (Scale bar, 100 µm.) (*C*) H&E staining of skeletal muscle cryosections, gastrocnemius (*top*), and diaphragm (*bottom*). (Scale bar, 50 µm.) (*D*–*G*) Absolute force produced by control and β-DG^cyto^ EDL muscles (*D*) and raw data from EDL muscles that performed an isometric tetanic contraction for 0.3-s (*E*). (*F*) EDL isometric force normalized to the estimated cross-sectional area of EDL muscles. (*G*) EDL force production following two eccentric contractions is shown as a percentage of force compared to the initial contraction. All data depicted in the figure were from male and female mice that were 13 to 18 wk of age. Data expressed as mean ± SD. *P*-values determined by the unpaired *t* test with Holm–Sidak post hoc analysis. *** = 0.0001; **** < 0.0001.

Murine DG consists of 893 amino acids (orthologous to 895 amino acids in humans) with residues 1 to 651 contained within α-DG and 652 to 893 within β-DG (Fig. 1*A*). Multiple domains across DG contribute to its function, including the signal peptide domain (residues 1 to 27), the α-DG N-terminal domain, consisting of an Ig-like domain (residues 61 to 161), a peptidase S72 domain (residues 180 to 301), and a mucin-like domain (residues 314 to 483). The α-DG C-terminal domain consists of an Ig-like domain (residues 496 to 600) and a peptidase S72 domain (residues 601 to 651) that is shared with β-DG N-terminal domain (residues 652 to 710). The remainder of β-DG consists of the transmembrane domain (residues 748 to 773), an intracellular juxtamembrane region (residues 774 to 878), and a carboxy-tail (residues 879 to 893).

The physiological importance of DG is revealed through genetic mutations that disrupt DG function and result in diseases referred to as dystroglycanopathies. These diseases manifest in various forms of limb-girdle muscular dystrophies (LGMDs), which can be accompanied by abnormalities of the eye and brain (14–16). Most of the dystroglycanopathies are due to genetic disruption of glycosyltransferases responsible for *O*-mannosylation of α-DG and formation of matriglycan, leading to either the absence of matriglycan or a shortened form of matriglycan, impairing the ECM-binding capabilities. Those diseases are termed secondary dystroglycanopathies, whereas primary dystroglycanopathies refer to genetic mutations within the *DAG1* gene. Only a few clinical cases of primary dystroglycanopathies with homozygous and heterozygous missense mutations within α-DG and β-DG subunits have been reported, with patient phenotypes ranging from early-onset severe muscular dystrophy with cognitive impairment (17–20) to childhood-onset with mild muscular dystrophy (21), as well as a late-onset form of limb-girdle muscular dystrophy (22). Two mouse models have been generated based on the missense mutations causing human disease, the T190M (orthologous to the human T192M) (18), and the C667F (orthologous to the human C669F) (23). As with the human patients, these mutations in the murine models resulted in skeletal muscle disease. Further insights into the function of DG in skeletal muscle were gleaned from mouse models harboring either a *Dag1* deletion (24–28) or deletion of α-DG N-terminus (α-DGN) (29, 30). Although valuable evidence has been gathered from assessing patients and murine models, most of what we know about DG in skeletal muscle is derived from *Dag1* deletion mouse models and mutations of residues within α-DG or the extracellular domain of β-DG. Hence, we have a poor understanding of the importance of intracellular β-DG in skeletal muscle.

The intracellular region of β-DG spans from residues 774 to 893. While numerous proteins are predicted to interact with β-DG (31), dystrophin is the most notable binding partner. Our lab previously determined that within in vitro cell lines, the cysteine-rich domain of dystrophin binds to a proline-rich region in the last 15 amino acids of the carboxy-terminal tail of β-DG (10). Structural analysis has further revealed that the interaction between β-DG and dystrophin can occur through linkage between the dystrophin’s WW and EF-hand domains (8, 11), and dystrophin’s ZZ domain (9). Additionally, studies have demonstrated that phosphorylation in the cytoplasmic region of β-DG and CR domain of dystrophin may regulate their interaction (32, 33). Collectively, these findings suggest that the interaction between dystrophin and β-DG is dynamic and can vary under different physiological conditions.

In skeletal muscle, DG is found at the sarcolemma and at the postsynaptic membrane of the neuromuscular junction (NMJ) (*SI Appendix*, Fig. S1*B*). Within the NMJ, α-DG binds agrin in the synaptic ECM, while β-DG interacts with utrophin and rapsyn in the subsynaptic membrane (12, 13, 34–41). Utrophin, the homolog of dystrophin, interacts with β-DG to establish the utrophin glycoprotein complex (UGC), primarily at the NMJ (12). The UGC is thought to be involved in the organization of the NMJ (42).

Rapsyn is concentrated at the synapse and appears to play a specialized role in clustering acetylcholine receptors (AChRs) and other postsynaptic proteins, helping establish synaptic size and synaptic AChR density (43, 44). The Cohen lab demonstrated that the RING-H2 domain of rapsyn interacts with the cytoplasmic region of β-DG (35). Our lab showed that rapsyn can cluster DG in muscle cell culture models, and this interaction does not rely on AChRs (13). Therefore, it is believed that rapsyn serves as the molecular link connecting DG to AChRs at the NMJ. Interestingly, evidence indicates that utrophin is required for localization of α-syntrophin and α-dystrobrevin at the postsynaptic membrane (45, 46). Although utrophin and dystrophin are not required for rapsyn to interact with β-DG (13), utrophin facilitates the interaction between rapsyn and α-dystrobrevin (46). Collectively, these data suggest that β-DG, utrophin, and rapsyn contribute to organizing the postsynaptic membrane.

Although the previous studies (12, 13, 35) demonstrated an interaction between DG and rapsyn and utrophin, the importance of these interactions in neuromuscular function has not been studied. Here, we examined the underlying importance of these interactions by generating and characterizing mouse models that lack portions of the cytoplasmic region of β-DG that mediate these interactions. We demonstrate that the cytoplasmic region of β-DG is necessary to organize the postsynaptic membrane, anchor AChRs, and ensure efficient neuromuscular function, essential for skeletal muscle force production and motor performance. These mouse models represent highly valuable tools to expand our mechanistic understanding of the in vivo functions of β-DG.

## Results

### The Intracellular Region of β-DG Is Required for Skeletal Muscle Function.

We generated mice in which the final 117 of 120 amino acids of the cytoplasmic region of β-DG were deleted (previously described by Satz et al. (47); Fig. 1*A* and *SI Appendix*, Fig. S2 *A* and *B*), and we refer to these mutant mice as β-DG^cyto^. As stated by Satz et al. (47), the frequency of homozygous β-DG^cyto^ mice did not fall within the expected range of allelic distribution, with only 9% of the offspring being identified as β-DG^cyto^ (*SI Appendix*, Fig. S2*C*). Further investigation determined that an 11.3% of β-DG^cyto^ mice died prior to embryonic day 7.5 (*SI Appendix*, Fig. S2*D*), suggesting that the mutant mice likely died due to malformation of Reichert’s membrane, as has been reported in DG-null mice (24). Because β-DG^cyto^ mice only have DG residues 1 to 776, skeletal muscle from these mice did not display immunofluorescent reactivity to antibodies that recognize the final 15 amino acids of the carboxy terminus tail of β-DG (Fig. 1*B*), including the affinity-purified rabbit antibody, AP83, and the 7D11 mouse antibody. However, immunofluorescent analysis of skeletal muscle revealed that matriglycan was present in the β-DG^cyto^ mice, although at a visibly lower intensity than that observed in control muscle (Fig. 1*B*). We then examined the localization of DGC proteins through immunofluorescence on transverse cryosections of the gastrocnemius muscles of control and β-DG^cyto^ mice (Fig. 1*B*). Control muscles displayed continuous sarcolemma and subsarcolemma localization of dystrophin and the sarcoglycans (β and γ) (Fig. 1*B*). Interestingly, DGC proteins were observed at or near the sarcolemma in muscle from β-DG^cyto^ mice (Fig. 1*B*). Muscle from young 3 to 4-wk-old β-DG^cyto^ mice, an age when the mutant mice would have endured a limited amount of fiber degeneration and regeneration, revealed similar observations as the adult mice (*SI Appendix*, Fig. S3*A*). Together, these data confirm that the loss of the cytoplasmic region in β-DG in skeletal muscle leads to disruption of the DGC, while retaining linkage of α-DG to the ECM.

Histological analysis of gastrocnemius and diaphragm muscles revealed dystrophic pathology, including variable fiber size and inflammatory cell infiltration (Fig. 1*C*). Thus, the loss of intracellular β-DG in skeletal muscle alters muscle fiber size and promotes muscle histopathology. We next assessed whether the absence of intracellular β-DG in skeletal muscle affected muscle function. We dissected extensor digitorum longus (EDL) muscles from control and β-DG^cyto^ mice for ex vivo examination of their contractile properties (Fig. 1 *D*–*G*). Maximal isometric tetanic contractile force and force per EDL cross-sectional area (i.e., specific force) were significantly lower in β-DG^cyto^ mice than in age- and gender-matched control mice (Fig. 1 *D*–*F*). We next assessed whether EDL muscles were susceptible to repeated stressors. Unlike EDL muscles from control mice, β-DG^cyto^ muscles could not maintain force production following repeated lengthening contractions (Fig. 1*G*). After only two lengthening contractions, β-DG^cyto^ muscles only produced approximately 20% of their initial contraction (Fig. 1*G*). These findings reveal that β-DG^cyto^ skeletal muscle fibers are weaker than those of age- and gender-matched controls and susceptible to functional impairment following forceful contractions, indicating that intracellular β-DG is required for skeletal muscle function.

### The Intracellular Region of β-DG Supports Formation of the Postsynaptic Terminal and Neuromuscular Function.

**Fig. 2.**
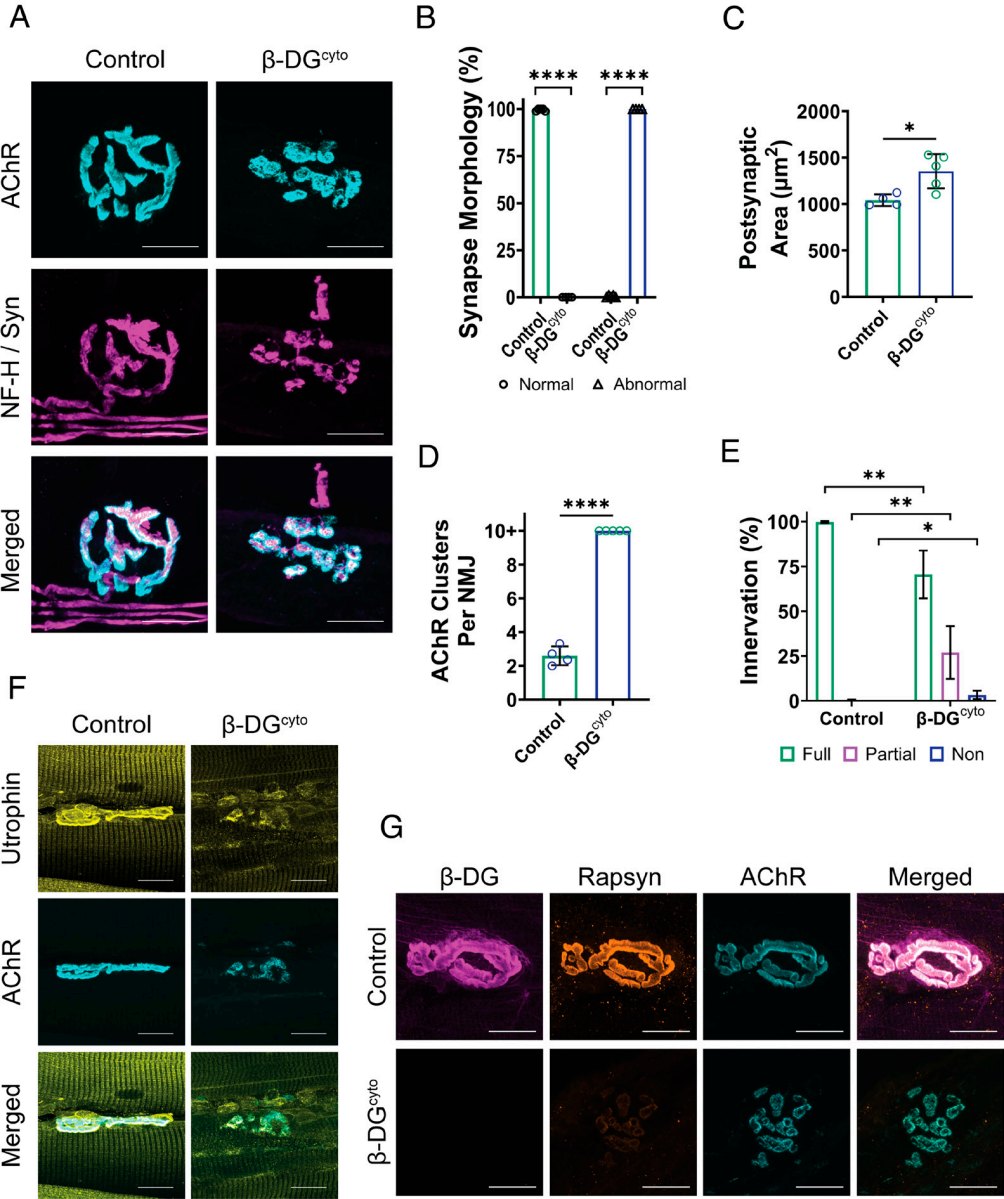
Deletion of the cytoplasmic region of β-DG disrupts the NMJ. (*A*–*E*). NMJ morphology in skeletal muscle from 15 to 20-wk-old control and β-DG^cyto^ mice. (*A*) Postsynaptic AChRs were labeled with alpha-bungarotoxin conjugated Alexa-fluor, and NF-H with Syn antibodies were used to label the motor axons and presynaptic terminal. (Scale bar, 20 µm.) (*B*) Determination of normal vs. abnormal postsynaptic morphology. Data reported as mean ± SD. **** < 0.0001. (*C*) Quantification of postsynaptic area. Data expressed as mean ± SD. * = 0.0156. (*D*) Number of discrete AChR-rich clusters at each synapse. Ten or more clusters per NMJ were grouped as 10+, as shown on the y-axis. Data reported as mean ± SD. **** < 0.0001. (*E*) Percentage of postsynaptic terminals that were innervated, partially innervated, or denervated in control and β-DG^cyto^ skeletal muscle. Data expressed as mean ± SD. Full ** = 0.0016; Partial ** = 0.0044; Non * = 0.0145. For all statistical analyses, unpaired *t* test with Holm–Sidak post hoc analysis were performed. (*F*) Immunofluorescence detection of utrophin and AChRs in EDL muscle from adult mice. (Scale bar, 20 µm.) (*G*) Immunofluorescence detection of postsynaptic β-DG, rapsyn, and AChRs in EDL muscle from adult mice. (Scale bar, 20 µm.)

In vitro studies have indicated that intracellular β-DG plays a role in the organization of the postsynaptic membrane at the NMJ (13, 34, 35, 40). Therefore, we examined how the absence of cytoplasmic β-DG influences the organization and function of the NMJ in an in vivo setting. First, we examined the morphology of NMJs from control and β-DG^cyto^ skeletal muscles (Fig. 2 *A*–*E* and *SI Appendix*, Fig. S3*B*). AChRs were labeled with alpha-bungarotoxin, while motor axons and the presynaptic terminals were labeled with antibodies to neurofilament H (NF-H) and synaptophysin (Syn) (Fig. 2*A* and *SI Appendix*, Fig. S3*B*). NMJs from control muscles displayed the expected pretzel-like morphology that was usually found as one to three continuous AChR-stained areas (Fig. 2 *A*, *B*, and *D*). In contrast, NMJs from β-DG^cyto^ muscles appeared abnormal, as the AChR-rich area was fragmented into multiple islands spread out over a modestly larger area (Fig. 2 *A*–*D*).

We then evaluated whether the observed abnormalities of the β-DG^cyto^ NMJs altered skeletal muscle innervation. Control muscles were found to have nearly 100% of the postsynaptic sites fully innervated, with less than 1% of the postsynaptic membrane being partially innervated (Fig. 2*E* and *SI Appendix*, Fig. S4*A*). However, a glaring disparity was observed in muscles from β-DG^cyto^ mice, as approximately 70% of synaptic sites were fully innervated while approximately 25% of synapses were partially innervated, and the remainder appeared fully denervated (Fig. 2*E* and *SI Appendix*, Fig. S4*A*). Obvious NMJ disruption was also observed in skeletal muscle from young 3 to 4-wk-old β-DG^cyto^ mice (*SI Appendix*, Fig. S3*B*), suggesting that NMJ disruption is due to absence of cytoplasmic β-DG rather than due to repeated cycles of muscle degeneration and regeneration.

Next, we examined whether intracellular β-DG is necessary for postsynaptic localization of utrophin (Fig. 2*F*) and rapsyn (Fig. 2*G*). First, we confirmed the presence of β-DG at the postsynapse in young (*SI Appendix*, Fig. S3*C*) and adult control mice (Fig. 2*G*), whereas muscles from young and adult β-DG^cyto^ mice lacked cytoplasmic β-DG (Fig. 2*G* and *SI Appendix*, Fig. S3*C*). While dystrophin has predominately been observed along the sarcolemma, utrophin has primarily been detected at the postsynaptic membrane at the NMJ (12, 42, 48). Like dystrophin, utrophin is a critical component of the UGC. Utrophin appeared poorly expressed and disorganized in and around NMJs in muscles from β-DG^cyto^ mice compared to muscle from control mice (Fig. 2*F*), suggesting that cytoplasmic β-DG is required for utrophin interaction and UGC assembly at the NMJ. Next, we examined the localization of rapsyn. While rapsyn colocalized with AChRs in control muscles, rapsyn appeared to be poorly expressed with AChRs in muscles from adult and young β-DG^cyto^ mice (Fig. 2*G* and *SI Appendix*, Fig. S3*D*). Together, these data indicate that intracellular β-DG participates in organizing the postsynaptic membrane, in part through its intracellular interaction with utrophin and rapsyn, suggesting that disrupting this organization contributes to impaired muscle innervation and function.

**Fig. 3.**
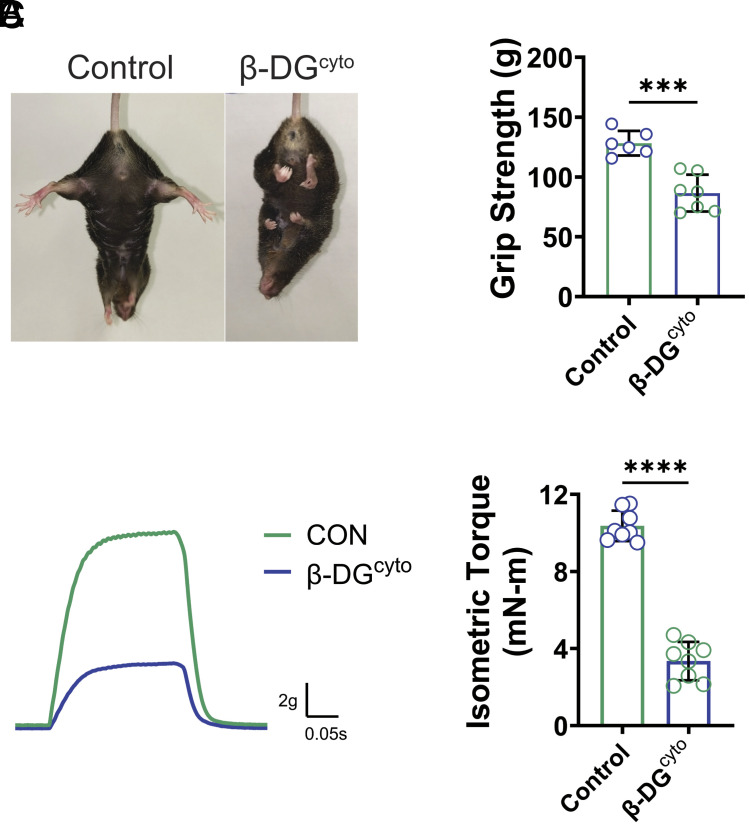
The cytoplasmic portion of β-DG is required for neuromuscular function. (*A*) Four-limb clasping observed in β-DG^cyto^ mice during tail suspension. (*B*) Forelimb grip strength from 12-wk-old mice. Data expressed as mean ± SD. (*C*) Raw data from in vivo electrical stimulation of plantar flexor skeletal muscles to induce an isometric tetanic contraction at 125 Hz for 0.2-s. Analysis of isometric torque production with the ankle positioned at 90° in mice aged 15 to 18 wk, shown in the graph. *P*-values determined by the unpaired *t* test with Holm–Sidak post hoc analysis. *** = 0.0002; **** < 0.0001.

Due to the structural disruptions of the NMJ from β-DG^cyto^ mice, we sought to evaluate in vivo neuromuscular function through various tests in control and β-DG^cyto^ mice (Fig. 3). Neuromuscular deficits were observed in the β-DG^cyto^ mice, as they displayed clasping of all four limbs (Fig. 3*A*), a characteristic often associated with neuromotor diseases (49–51). At 12 wk of age, forelimb grip strength was significantly lower in β-DG^cyto^ mice than in age- and gender-matched controls (Fig. 3*B*). In vivo analysis of the plantar flexor muscles in 15 to 18-wk-old mice revealed that the β-DG^cyto^ mice produced lower torque output from nerve-stimulation than those of control mice (Fig. 3*C*). These data demonstrate that the cytoplasmic region of β-DG has an important role in regulating neuromuscular strength and motor performance, potentially through disruption of the organization of the postsynaptic membrane.

### Deletion of the Carboxy Terminal Tail of β-DG Does Not Lead to Skeletal Muscle Dysfunction or Neuromuscular Disease.

**Fig. 4.**
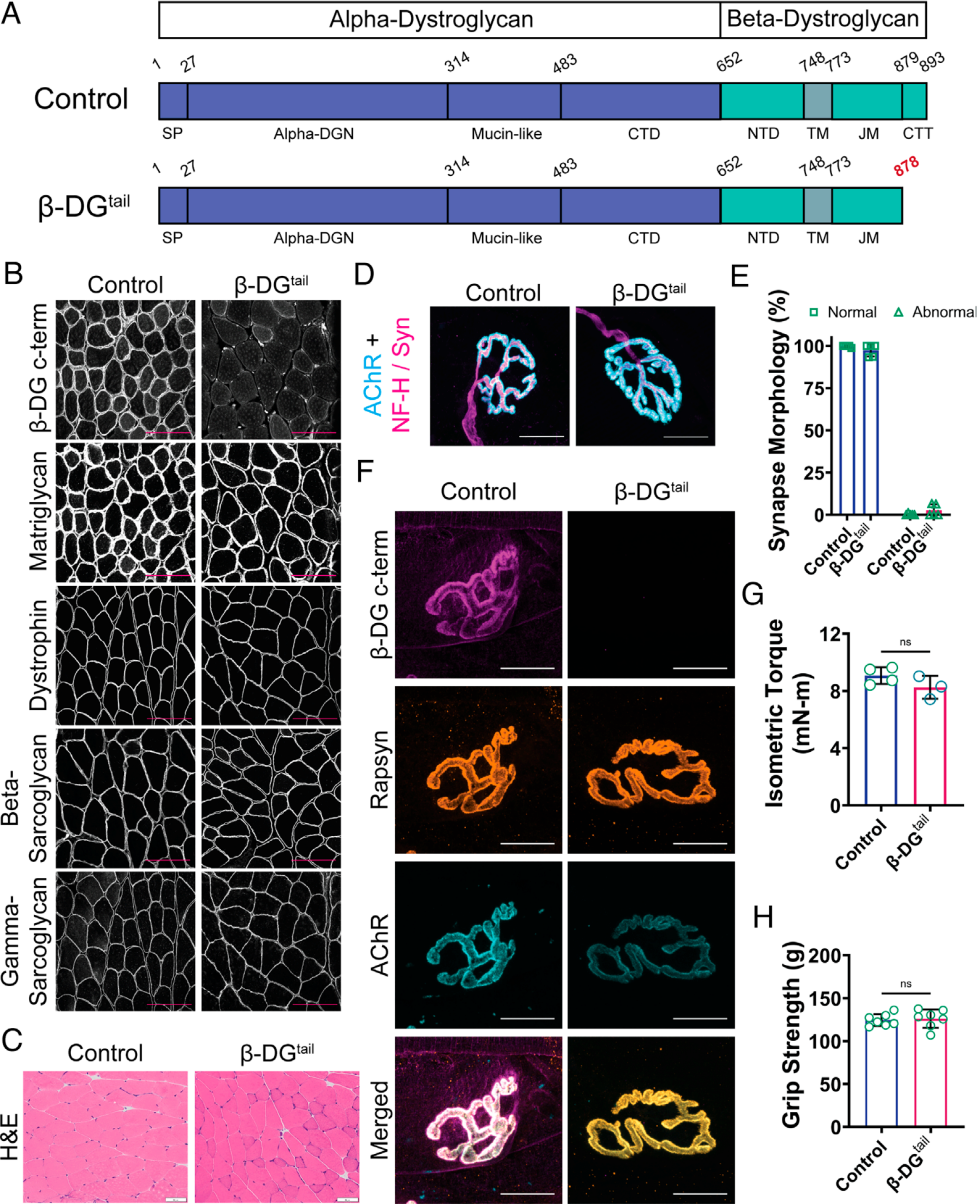
Deletion of the carboxy-tail of β-DG does not lead to neuromuscular pathology. (*A*) Depiction of control and β-DG^tail^ alleles demonstrating the lack of the final 15 residues of β-DG (ending at residue 878, as indicated by red font) in the β-DG^tail^ mice. (*B*) Immunofluorescence of gastrocnemius muscle cryosections probing for the carboxy-tail of β-DG with the antibody 7D11 (*top* *row*) and matriglycan, the *O*-mannosyl modification on α-DG responsible for binding to ECM ligands, with the antibody IIH6 (*second row*); dystrophin (*third row*); β-Sarcoglycan (*fourth row*); and γ-Sarcoglycan (*bottom* *row*). (Scale bar, 100 µm.) (*C*) H&E staining of gastrocnemius muscle cryosections. (Scale bar, 50 µm.) (*D*) Detection of the NMJ with alpha-bungarotoxin to detect AChRs (*cyan*) and NF-H plus Syn to detect the motor axon and presynaptic terminal in EDL muscle. (Scale bar, 20 µm.) (*E*) Determination of normal vs. abnormal postsynaptic morphology. (*F*) Immunofluorescence of postsynaptic β-DG, rapsyn, AChR, and a final merged image. (Scale bar, 20 µm.) (*G*) Isometric torque production with the ankle positioned at 90° in mice aged 15 to 18 wk, shown in the graph. (*H*) Forelimb grip strength from 12-wk-old mice. Data expressed as mean ± SD. *P*-values determined by the unpaired *t* test with Holm–Sidak post hoc analysis.

Our lab previously demonstrated that the proline-rich, carboxy tail consisting of the final 15 amino acids of β-DG could bind to dystrophin within an in vitro cell culture model (10). Therefore, we attempted to narrow down the intracellular region of β-DG vital for muscle function. In order to determine whether the carboxy tail of β-DG is essential for skeletal muscle function, we generated mice in which the final 15 amino acids of β-DG were deleted (Fig. 4*A* and *SI Appendix*, Fig. S5 *A* and *B*), and we refer to these mice as β-DG^tail^. The β-DG^tail^ mice were viable, and the mutant mice were fertile. Immunofluorescent analysis confirmed that β-DG^tail^ skeletal muscle tissue lacks the carboxy tail of β-DG while retaining the connection to α-DG and the ability to form matriglycan on the mucin-like domain of α-DG (Fig. 4*B*). Histological analysis of gastrocnemius muscles revealed that β-DG^tail^ muscle appeared healthy without overt signs of disease (Fig. 4*C* and *SI Appendix*, Fig. S6*A*). Additional findings demonstrate that DGC formation (Fig. 4*B*), ex vivo skeletal muscle force production (*SI Appendix*, Fig. S6*B*), and the ability to withstand repeated eccentric contractions (*SI Appendix*, Fig. S6*C*) are not reliant on the carboxy tail of β-DG. Last, we demonstrate that the carboxy tail of β-DG is not essential for organization of the postsynaptic membrane (Fig. 4 *D*–*F*) or neuromuscular function (Fig. 4 *G* and *H*).

## Discussion

Although in vitro evidence suggests multiple important roles for the intracellular region of β-DG, our understanding of this region in an in vivo environment is limited. In the present study, we provide evidence that the cytoplasmic region of β-DG is necessary for skeletal muscle force production, AChR clustering and stability at the NMJ, thus proving that this domain is vital for neuromuscular function and overall health.

Previous observations from our laboratory demonstrated that DG is essential for the formation of Reichert’s membrane during murine embryonic development (24). Whereas our earlier study utilized *Dag1* deletion, resulting in deletion of both α- and β-subunits, in the present study, we utilized mice that lacked the final 117 amino acid residues of cytoplasmic β-DG (β-DG^cyto^). These mice were predicted to have only four intracellular residues below the plasma membrane (47). As we previously reported (47), approximately 10% of β-DG^cyto^ offspring survived in utero development, rather than the expected 25%. Moreover, our current analysis determined that loss of approximately 15% of β-DG^cyto^ offspring occurred prior to embryonic day 7.5, thus in accordance with the observations from *Dag1*-deficient mice that did not survive past embryonic day 7 (24). Partially penetrant embryonic lethality was recently reported in a mouse model containing a point mutation C667F within the extracellular portion of β-DG (23). However, the C667F point mutation led to death after embryonic day 10.5 (23). While these data emphasize the importance of an undisturbed DG subcomplex in murine development, embryonic lethality has also been observed in mouse models of secondary dystroglycanopathies (52–56), which led to the development of conditional knockout (KO) mouse lines (55, 57–60). In dystroglycanopathy mouse lines that display partial penetrance embryonic lethality, the mice that survive birth ultimately display signs of muscular dystrophy (23, 56), similar to the β-DG^cyto^ mice.

In the present study, surviving β-DG^cyto^ mice displayed multiple skeletal muscle dystrophic characteristics, including histopathology (i.e., variable muscle fiber size, centrally located nuclei, and inflammatory cell infiltration), accompanied by impaired contractile function, and repeated forceful contractions exacerbated the contractile impairment. Disruption of DG or other proteins within the DGC can destabilize the entire complex, leading to skeletal muscle weakness and increased susceptibility to eccentric contraction-induced reductions in force (5, 61–64). In the present study, β-DG^cyto^ muscle displayed reduced expression of matriglycan, sarcoglycans, and dystrophin at or near the sarcolemma. The functional deficits reported in the skeletal muscle from the β-DG^cyto^ mice appear to be comparable, if not greater than those routinely reported for the dystrophin-deficient *mdx* mice (65, 66). Although muscle from both the β-DG^cyto^ and *mdx* mice have reduced protein content of DGC components (67, 68), evidence from *mdx* mice indicates utrophin can be upregulated and compensates for the absence of dystrophin, thereby limiting the severity of muscle fragility and dysfunction (69, 70). Whereas the truncated β-DG in the β-DG^cyto^ muscle prevents the interaction between utrophin and β-DG, which may contribute to their severe muscle weakness and greater vulnerability to lengthening contraction-induced damage. These findings demonstrate that intracellular β-DG is required for the proper assembly and stability of the DGC, which is necessary for functional transmission of force in muscle. However, several mechanisms likely contribute to the skeletal muscle phenotype of the β-DG^cyto^ mice.

Beyond the sarcolemma-localized DGC, DG is also found at the postsynaptic membrane at the NMJ (13, 25, 40, 71–73). A distinctive trait of the NMJ is the high-density clustering of AChRs at the postsynaptic membrane, essential for efficient synaptic transmission (74–76). DG is among a few of the DGC proteins that participate in the maturation and maintenance of AChR clustering at the NMJ. While earlier studies attributed AChR clustering abnormalities in muscular dystrophy to cycles of muscle fiber degeneration and necrosis (77, 78), more recent studies demonstrated that abnormal AChR clustering is a consequence of DGC disruption (79). At the NMJ, β-DG is associated with utrophin, dystrophin, and rapsyn (12, 13, 34, 35, 80, 81), while extensively glycosylated α-DG interacts with laminins and agrin in the extracellular space (37, 39, 82). Evidence indicates that both α- and β-DG play important roles in the clustering of AChRs in cultured myotubes and the formation and maturation of NMJs in vivo (13, 25, 36–40, 72, 73, 83–86). For example, DG-null cultured myotubes display disorganized, unstable, and highly dispersed AChR clusters (72). Furthermore, chimeric DG-deficient mouse skeletal muscle fibers displayed an aberrant NMJ morphological phenotype (25). Although it is evident that DG is an important component of the NMJ, the contribution of intracellular β-DG to the NMJ is lacking. Poorly formed NMJs have previously been noted in *mdx* (77, 78, 87), utrophin KO mice (42, 48), and the double KO that is deficient in utrophin and dystrophin (69, 70). In our present study, we show that postsynaptic intracellular β-DG is necessary for the synaptic localization of utrophin and rapsyn and provide data supporting the requirement of DG for AChR clustering. Together, these data support predictions that DG assists in organizing AChRs at the NMJ. While mice that lack agrin, low-density lipoprotein receptor-related protein 4 (Lrp4), muscle-specific kinase (MuSK), Dok-7, and rapsyn fail to form neuromuscular synapses and consequently die from neonatal lethal respiratory failure (43, 88–90). β-DG^cyto^ mice developed poorly formed NMJs and were capable of surviving to adulthood. Thus, it is likely that low levels or partially functional DG and associated protein complexes are sufficient for imperfect synaptogenesis.

Due to the observed NMJ disruption in the β-DG^cyto^ muscles, we investigated whether these disruptions were associated with neuromuscular dysfunction. A lack of intracellular β-DG led to four-limb clasping, lower levels of plantar flexor torque, and reduced forelimb grip strength. These data provide in vivo evidence for intracellular β-DG in organizing the postsynaptic membrane, through which it can influence neuromuscular function. Utrophin KO, *mdx*, and *mdx*/utrophin double KO mice have poorly formed but moderately functional NMJs with an age-associated functional decline (42, 48, 69, 70, 77, 87). While we observed reduced nerve-stimulated muscle strength and grip strength in the β-DG^cyto^ mice, we cannot rule out the influence that severely diseased skeletal muscle, rather than or in addition to disorganization of the NMJ, plays in muscle strength. Furthermore, although morphological changes observed in the NMJs from β-DG^cyto^ mice are clearly indicative of an altered state, our current study lacks data on neurotransmission. The functional correlates of the structural abnormalities observed at the synapses in β-DG^cyto^ skeletal muscle are unknown and may not necessarily indicate a reduced ability to transmit an action potential. Therefore, the overall significance of the postsynaptic disruption remains unknown. Importantly, data from our β-DG^cyto^ mice and utrophin KO, *mdx*, and double KO mice emphasize the resilience of the NMJ and its ability to function in response to disruption. Interestingly, increasing expression of Lrp4, an essential component of the NMJ, in dystrophin-deficient *mdx* mice led to reduced NMJ fragmentation, less denervation, and improved neuromuscular transmission (91). In view of those findings, it is possible that therapeutic strategies designed to improve attachment of nerve terminals (92–95) may be an effective approach to limit neuromuscular functional decline that accompanies myopathologies due to disruption of the DGC. Altogether, these observations emphasize the importance of DG and the DGC in neuromuscular function.

We have previously shown that the carboxy-terminal tail of β-DG, a proline-rich domain comprising 15 residues, can bind to dystrophin within in vitro cell models (10). This observation led us to develop mice that lacked the carboxy-tail of β-DG (β-DG^tail^), allowing us to determine whether this C-terminal segment is vital for skeletal muscle development and function. Despite the absence of the carboxy-tail domain, β-DG^tail^ mice were viable, displayed matriglycan levels similar to those of controls, and their skeletal muscle was not observably pathological. Furthermore, β-DG^tail^ muscle displayed similar levels and localization of DGC proteins as those noted in the muscle from control mice. Based on the intact DGC and lack of histopathology, it was unsurprising that β-DG^tail^ skeletal muscles were capable of producing forceful contractions and able to withstand multiple eccentric contractions without a reduction in force like that seen in muscle from β-DG^cyto^ mice. Additionally, the β-DG^tail^ mice developed and maintained the pretzel-like morphology of the postsynaptic terminals and displayed neuromuscular function similar to control mice. Taken together, these findings indicate that assembly of the DGC, postsynaptic organization, and neuromuscular function are not reliant on the carboxy-tail domain of β-DG. Observations from a knock-in DG mouse model harboring a mutation of residue 890 from tyrosine to phenylalanine did not cause a dystrophic phenotype, even in mice up to 8 mo old (96). Although the Y890F *Dag1* mutation did not cause disease, phosphorylation of residue 890 was shown to promote proteasomal degradation of the DGC (96), thus providing functional insight into the C-terminal tail of β-DG. The present evidence indicates that the C-terminal tail of β-DG is sufficient to bind dystrophin but not necessary for membrane localization of dystrophin and skeletal muscle function, suggesting that dystrophin may interact with more than one region within the intracellular tail of β-DG. Consistent with this idea, recent cryo-EM analysis of the intact DGC revealed that a juxtamembrane fragment of β-DG (residues 780 to 794; orthologous to murine 778 to 792) directly interacts with dystrophin’s ZZ domain (9), raising the possibility that dystrophin may bind to both β-DG’s C-terminal tail and juxtamembrane domains. Unfortunately, the recent studies lack the resolution to elucidate the intracellular region of β-DG and its interactions fully (8, 9). Even less is known concerning the interaction between β-DG and dystrophin’s homolog, utrophin, and the exact residues underpinning this interaction have not been confirmed. The interaction between β-DG and rapsyn has been shown to occur between the RING-H2 domain of rapsyn (35) and the cytodomain of β-DG (residues 787 to 819) (97). Accordingly, these findings and those of the β-DG^cyto^ lead us to believe that the cytoplasmic region of β-DG is essential for skeletal muscle health and neuromuscular function.

In the current study, we utilized two mouse models as molecular tools for investigating intracellular β-DG. Although we provide examination of skeletal muscle from the β-DG^cyto^ mouse, this mouse model has been utilized previously in studies that were essential for expanding our knowledge of DG in the eye (47, 98) and brain (99–102). The present study also provides a description of the skeletal muscle phenotype from the β-DG^tail^ mouse model. It is important to note that both truncated β-DG mouse models used in this study are constitutive deletion models and are not skeletal muscle-specific. Therefore, we cannot discount the potential influence of truncated β-DG in nonskeletal muscle cell types on the phenotypes observed in the current study. For instance, *Dag1* is ubiquitously expressed throughout the body, including skeletal muscle, cardiac muscle, and the central and peripheral nervous systems (3, 26, 99, 103–105). In the peripheral nervous system, *Dag1* is expressed by myelinating Schwann cells, where it plays important roles in myelin sheath formation and maintenance, as well as in stabilizing sodium channels in the plasma membrane (104). Selective deletion of *Dag1* in Schwann cells has been shown to slow the conduction velocity of the electrical impulse in caudal and sciatic nerves, contributing to abnormal pain responses and impaired motor performance in mice (104). In cardiac muscle, DG provides structural support to the sarcolemma and transverse tubules (59, 105). The absence of functional DG in cardiac fibers leads to a progressive cardiomyopathy that can be triggered by cardiac stress (59, 105), eventually impairing the heart’s pumping action and delivery of blood throughout the body. Although the potential involvement of DG in skeletal muscle tissue vascularization remains poorly characterized, there is evidence of vascular abnormalities and impaired blood flow in dystrophin-deficient *mdx* mice (106, 107). However, potential functional ischemic events in skeletal muscle tissue may be due to impaired nitric oxide generation derived from within the muscle fibers (108). Ultimately, our constitutive deletion mouse models provide an opportunity to investigate the systemic importance of dystroglycan. Moreover, both of our mouse models allow for the examination of binding partners of intracellular β-DG within an in vivo setting. The unique mouse models will expand our understanding of primary dystroglycanopathies, as well as disorders resulting from disruption to β-DG binding partners. For instance, targeted therapies for dystrophin deficiency, clinically known as Duchenne muscular dystrophy and Becker muscular dystrophy, include gene editing strategies that result in the production of truncated and partially functional dystrophin (109). Therefore, designing the most effective dystrophin-targeted therapeutic strategies will require a complete understanding of the β-DG–dystrophin interaction.

In conclusion, we provide evidence that the intracellular region spanning residues 777 to 893 of β-DG is essential for skeletal muscle health and neuromuscular function and vital for preventing myopathology. We show that muscle function relies on this cytoplasmic region β-DG to support maturation and maintenance of the NMJ. These findings expand our molecular understanding of β-DG in vivo and further define the mechanisms underlying neuromuscular health.

## Materials and Methods

Detailed description of the care and use of animals, generation of β-DG deletion mouse models, and skeletal muscle and neuromuscular phenotyping procedures can be found in *SI Appendix*. In addition, a list of antibodies, descriptions of histology and immunofluorescence procedures, image capture and analysis, and a summary of statistical analyses are included in *SI Appendix*.

## Supplementary Material

Appendix 01 (PDF)

## Data Availability

All materials and reagents used in this study are detailed in the article and/or *SI Appendix*. Data have been deposited in figshare (110). All relevant data are included in the article and/or *SI Appendix*.
