# Supplementary material for: Cytoplasmic region of beta-dystroglycan is essential for postsynaptic maturation and neuromuscular function in mice
Source: Proc Natl Acad Sci U S A. 2026 Jun 3;123(23):e2600931123. doi: 10.1073/pnas.2600931123 (PMC13250547; doi:10.1073/pnas.2600931123)
Supplement: Supplementary file 1 — Appendix 01 (PDF) [file pnas.2600931123.sapp.pdf]

Supporting Information for

Cytoplasmic region of beta-dystroglycan is essential for postsynaptic maturation and neuromuscular function in mice

Jeffrey M. Hord[a,b,c,d], Rolf Turk[a,c,d], Hajime Kusano[a,c,d], Erik P. Rader[a,c,d,e], Sarah Burns[a,c,d], Zeita Gastel[a,c,d], Sally J. Prouty[a,c,d], Liping Yu[c,f,g], Steven J. Burden[h], Kevin P. Campbell[a,c,d,1]

Kevin P. Campbell, PhD
Email: kevin-campbell@uiowa.edu

This PDF file includes:

Figures S1 to S6
SI Materials and Methods
SI References

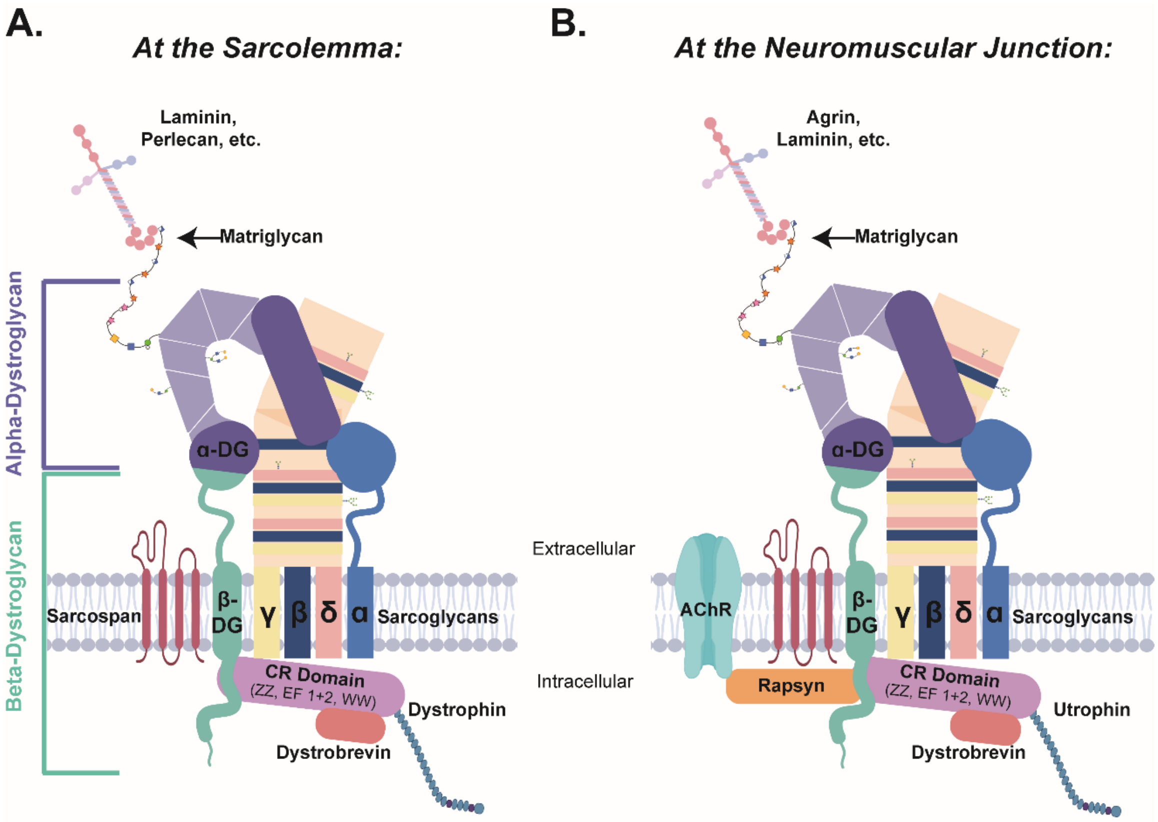

Fig. S1. Illustrations of dystroglycan within protein complexes and dystroglycan domains. **A.** An illustration of the dystrophin glycoprotein complex (DGC) is based on recent structural analyses of the complex (1, 2). The dystroglycan (DG) subcomplex is made up of the extracellular α-DG and β-DG, which spans from extracellular to intracellular. Through modification of the mucin-like domain of α-DG resulting in the heteropolysaccharide matriglycan, DG acts as a receptor for extracellular ligands such as laminin and agrin. The sarcoglycan subcomplex extends into the extracellular space through its sizeable extracellular domain tower comprised of intertwined β-, γ-, and δ-sarcoglycans. In the transmembrane region, β-DG is flanked by the sarcoglycans (α-, β-, γ-, and δ-) on one side and sarcospan on the opposing side. Intracellularly, dystrophin forms a subcomplex with dystrobrevin. The dystrophin subcomplex links to the sarcolemma proteins through the interaction of its cysteine-rich (CR) domain with the cytoplasmic region of β-DG and the sarcoglycans. **B.** Illustration of the utrophin glycoprotein complex (UGC). Central components are similar to the DGC except where noted. Dystrophin is replaced by its homolog utrophin. The cytoplasmic region of β-DG interacts with the postsynaptic protein rapsyn, and rapsyn provides a link to acetylcholine receptors (AChRs) in the postsynaptic membrane.

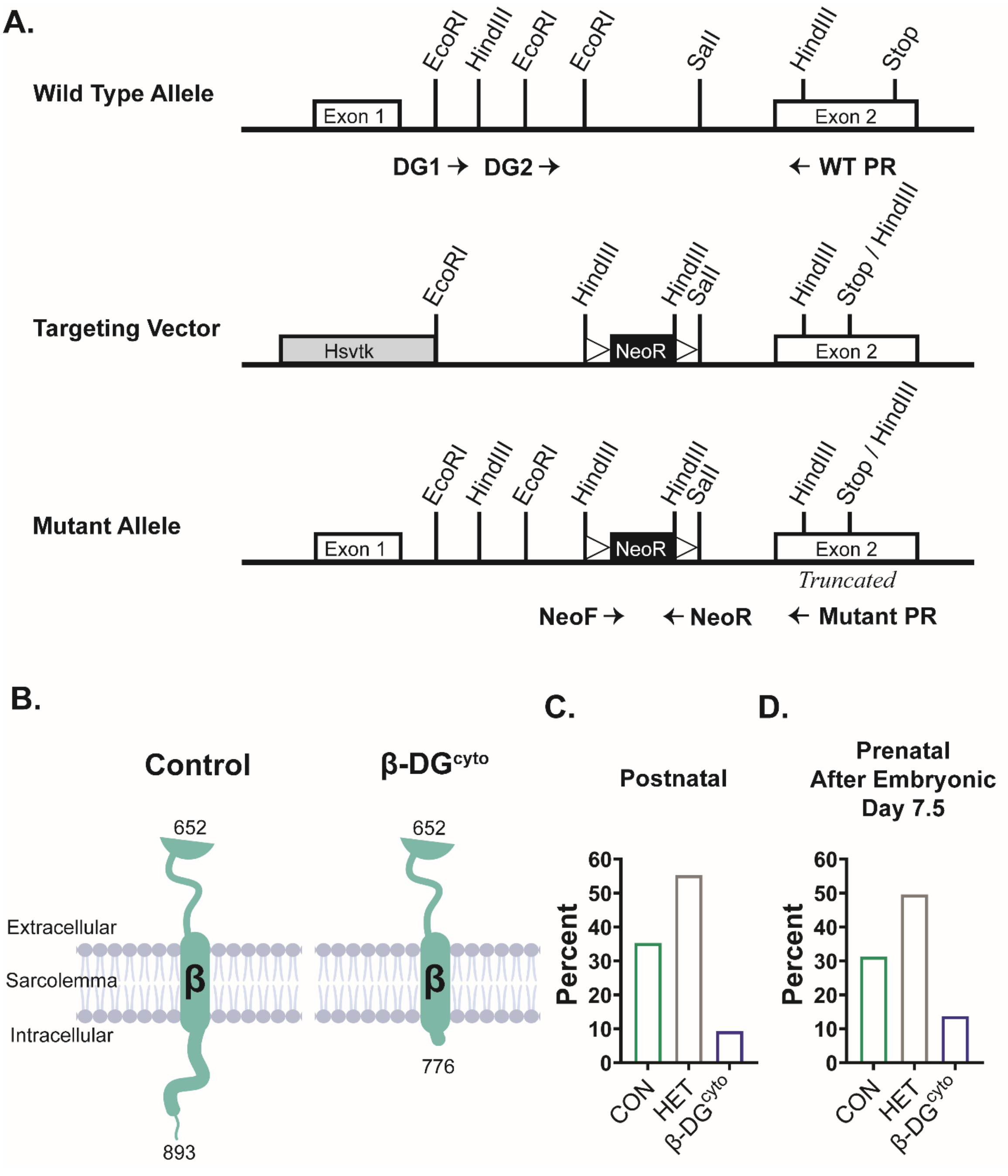

Fig. S2. Generation of β-DG^{cyto} mice. A. Targeting strategy for generation of β-DG^{cyto} mice (previously described (3)). **B.** Illustrations depicting β-DG in control and β-DG^{cyto} skeletal muscle fibers. The N-terminal residue and last residue of β-DG are shown for control and β-DG^{cyto} mice. **C-D.** Allelic distribution of litters from heterozygous (HET) male x HET female breeding. **C.** Genotype results from postnatal pups and (**D**) from embryos ranging from embryonic day 7.5 to 18.5.

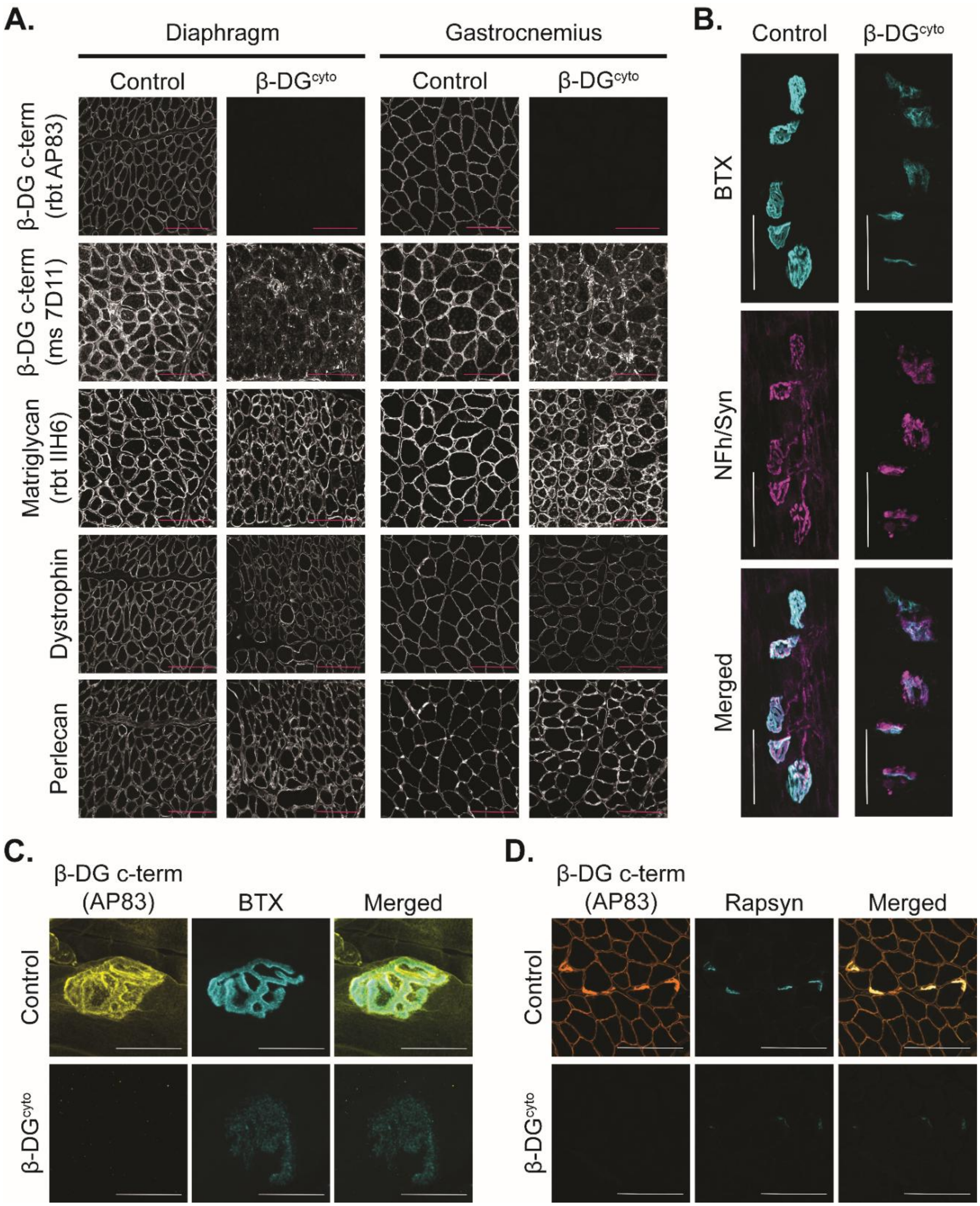

Fig. S3. Skeletal muscle fiber and post-synaptic pathology observed in young (3-4 weeks) β-DG^{cyto} mice. A. Immunofluorescence of diaphragm and gastrocnemius muscle cryosections probing for the carboxy-tail of β-DG (AP83 antibody in *top row*; 7D11 antibody in *second row*), matriglycan, dystrophin, and perlecan. Scale bars = 100 μm. **B.** Neuromuscular junction (NMJ) morphology in control and β-DG^{cyto} diaphragm muscle. Postsynaptic acetylcholine receptors (AChR) are labeled with α-bungarotoxin (BTX), and neurofilament H (NF-H) with synaptophysin (Syn) antibodies were used to label the motor neuron and presynaptic terminal. Scale bar = 100

μm. **C.** Fluorescent detection of post-synaptic β-DG and AChRs (BTX) in gastrocnemius muscle. Scale bar = 20 μm. **D.** Immunofluorescence detection of β-DG and rapsyn in gastrocnemius cryosections. Scale bar = 100 μm. All data depicted in panels **A-D** were from male and female mouse pups that were 3-4-weeks of age.

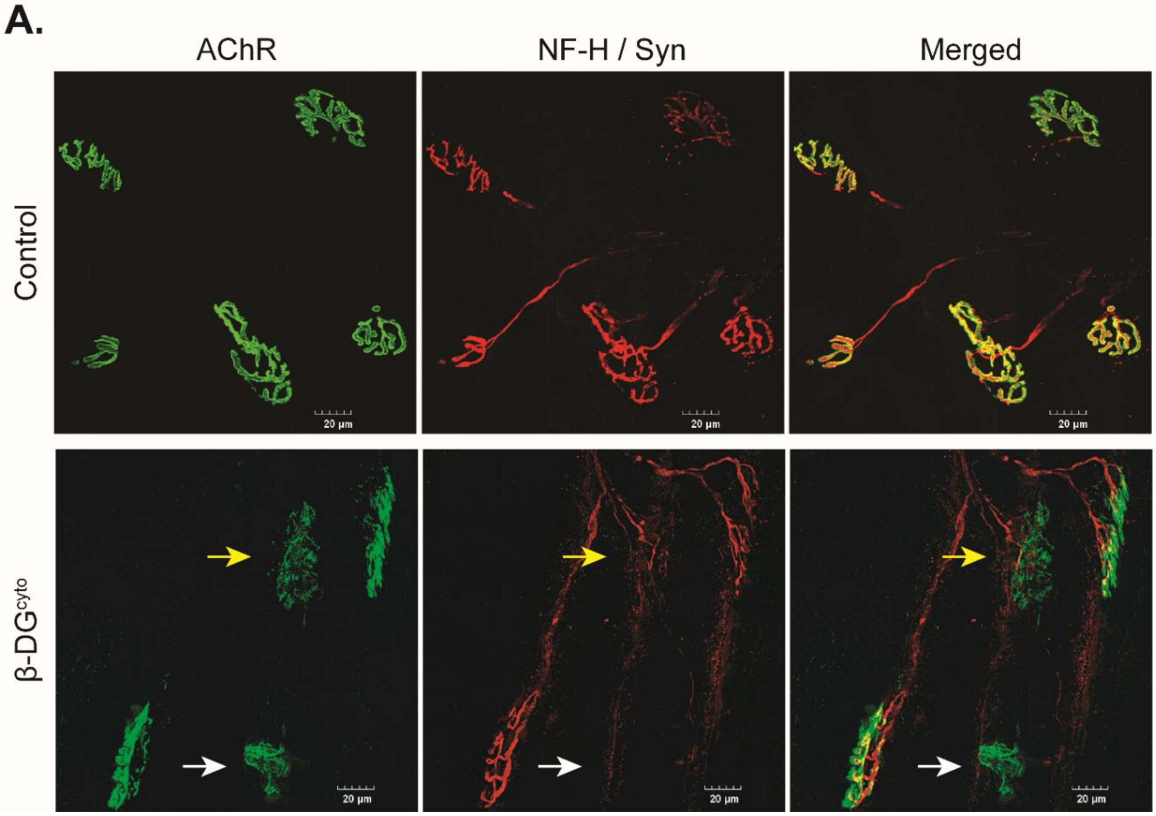

Fig. S4. Skeletal muscle fiber innervation in control and β-DG^{cyto} mice. A. Fluorescent images of neuromuscular synapses. Postsynaptic acetylcholine receptors (AChR) are labeled with α-bungarotoxin, and neurofilament-H (NF-H) with synaptophysin (Syn) antibodies are used to label the motor neuron and presynaptic terminal. Scale bar = 20 µm. Muscle from β-DG^{cyto} mice contained postsynapses that were partially innervated (yellow arrow) and denervated (white arrows).

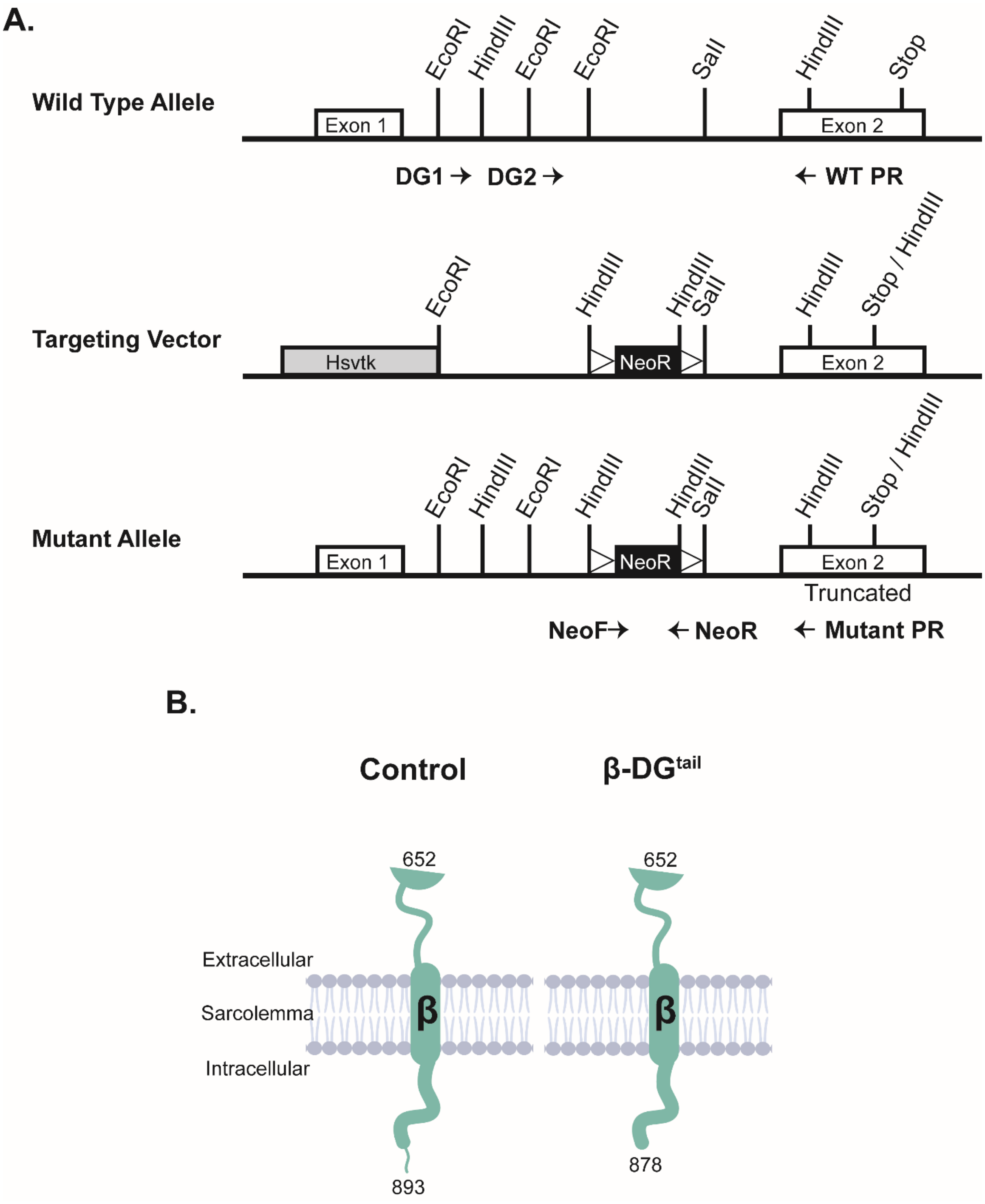

Fig. S5. Generation of β-DG^{tail} mice. A. Targeting strategy for generation of β-DG^{tail} mice. **B.** Illustrations depicting β-DG in control and β-DG^{tail} skeletal muscle fibers. The N-terminal residue and last residue of β-DG are shown for control and β-DG^{tail} mice.

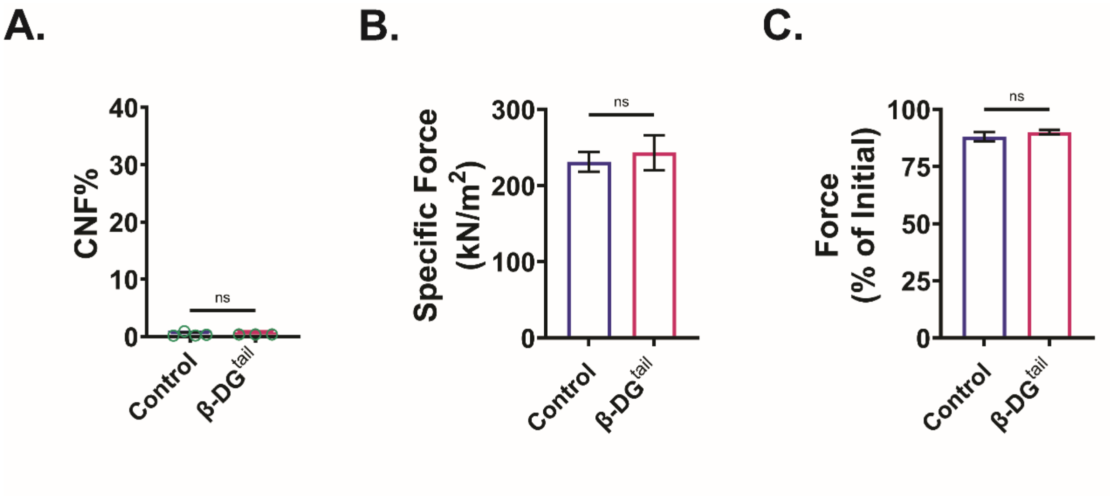

Fig. S6. β-DG^{tail} skeletal muscle characteristics. A. Total percentage of central nucleated myofibers (CNF) from diaphragm. **B.** EDL isometric force normalized to the estimated cross-sectional area of EDL muscles. N = 5 per group. **C.** EDL force production following two eccentric contractions is shown as a percentage of force compared to the initial contraction. N = 5 per group. All data depicted were from male and female mice that were 13-18-weeks of age. Data expressed as mean ± standard deviation. P-values determined by unpaired t-test with Holm-Sidak post-hoc analysis.

SI Material and Methods:

Animals

Animal care, ethical use, and procedures were carried out following protocols approved by the National Institutes of Health and the Institutional Animal Care and Use Committee (IACUC). Mice were housed socially whenever possible, except when single housing was necessary, in an Association for Assessment and Accreditation of Laboratory Animal Care (AAALAC)-accredited facility that maintains specific pathogen-free conditions. Mouse housing conditions adhered to the guidelines outlined in the Guide for the Care and Use of Laboratory Animals (National Research Council). A reverse 12h/12h light/dark cycle was implemented, and *in vivo* mouse assessments were conducted only during the dark cycle. Standard rodent chow (Harlan Laboratories, Indianapolis, IN, USA) and water were available *ad libitum*. Wild-type mice from the β-DG^{cyto} mouse line served as controls. When available, littermates were used as controls. Both male and female mice were used. Within each experiment, mice were matched by age and sex whenever possible. Group designations (randomization) were assigned according to identification numbers and genotype data prior to the experimenter observing the mice, ensuring no bias based on mouse phenotype. Due to the progressive decline in motor and respiratory functions with age, target mice were euthanized because of decreased body condition, inability to feed, paralysis of the hindlimbs, or respiratory distress, as dictated in our IACUC protocol. Animal use and data reporting adhered to the Animal Research: Reporting of *In Vivo* Experiments (ARRIVE) guidelines.

The generation and genotyping protocols for β-DG^{cyto} mice have been described previously (3). To generate the β-DG^{tail} mice, a 4.3 kb SalI-EcoRV fragment of mouse genomic dystroglycan gene (4) that includes exon 2 was subcloned into pBluescript KS (+) (pBSDGSE). PCR was carried out using primers H1 (5'CAAGCTCAAGCTGACCC3') and DBDR (5'AAGCTTTCACTCACTCAGGGACGGGAGCCCTTG3') and also DBDF (5'TGAGTGAGTGAAAGCTTCCCCATACCGATCACCC3') and P1 (5'CCCACCCATACCCTTTA3'). DBDR and DBDF incorporate stop codons in all three reading frames and a HindIII site as a marker for stop codons. The resulting PCR products were 1062 bp and 605 bp, respectively. The PCR products were mixed together and subjected to a second round of PCR using primers H1 and P1 only. The resulting product of 1643 bp was digested with internal NheI and PmlI sites and re-subcloned into pBSDGSE resulting in PBSDGSE-STOP which contained exon 2 of the dystroglycan gene with a premature stop codon and diagnostic HindIII site. To make the final targeting vector, a triple ligation was carried out using pBSDGfloxtk, pBSDGESTOP and EC1 creating a 17.5 kb plasmid pBSDGSTOP which had the premature stop codon, a neoR gene, and HSVtk promotor and enough flanking sequences to allow for homologous recombination. R1 ES cells were electroporated with 25 ug of linearized targeting vector and doubly selected with neomycin and ganciclovir. Surviving ES cell colonies were expanded and frozen down for DNA analysis. DNA prepared from surviving ES cell colonies was digested with HindIII and analyzed by Southern analysis using two probes. Probe A detected the presence

of the neo gene by detecting a decrease in the size of a HindIII fragment from 8.2 to 5.8 kb due to the introduction of a HindIII site along with the neo cassette. Probe B detected the additional HindIII site introduced along with the stop codons into the gene and resulted in a 1 kb fragment in correctly targeted cells, while cells without the stop codons would carry a wildtype band of 10.0 kb long. Of the 70 ES cell clones analyzed, 74% targeted to the dystroglycan gene. Of these, 63% (a total of 33) also displayed recombination 3' of the introduced stop codons resulting in correctly targeted ES cells. Two of these ES cells were grown and injected into recipient C57Bl/6 blastulas. Chimeric offspring, based on coat color were backcrossed to C57Bl/6 mice and offspring were genotyped. Chimeric mice were identified by a PCR assay in which either the wildtype sequence or the mutated sequence was detected using oligos directed against either the wildtype or the stop codon sequences. Heterozygous mutant mice from the F1 generation were crossed to obtain homozygous mutant mice.

Grip Strength

The forelimb grip strength experimental protocol was conducted in accordance with the procedures outlined in the TREAT-NMD standard operating protocol #DMD_M.2.2.001, unless otherwise specified. Mice were allowed to acclimate to the procedure room for 10 min. The murine grip strength meter (Columbus Instruments, Columbus, OH, USA) was horizontally mounted, with a nonflexible wire grid connected to the force transducer. Mice performed five pulls of the grid, while allowing a brief recovery between the third and fourth pulls. The mean of the three highest pulls was used to determine the average mouse grip strength.

***In Vivo* Plantar Flexion Torque**

Plantar flexor torque was performed as described previously (5), except where stated. Plantar flexion of the triceps surae was assessed with an *in vivo* muscle contractility device (Model 1300A, Aurora Scientific, Inc., Aurora, Ontario, Canada). Mice were anesthetized with inhaled isoflurane, administered at 3-4% for induction and 1.5-2.5% for maintenance (SomnoSuite Low-Flow Anesthesia System, Kent Scientific, Torrington, CT, USA). To prevent dryness of the eyes and corneal irritation, ophthalmic lubricating gel was applied to both eyes of each mouse. Mice were carefully laid in a supine position on a temperature-regulated testing platform set at 37°C. The right knee was stabilized with blunt clamps, and the right foot was securely fixed onto the footplate connected to the servomotor. The hind paw was secured to the force plate with adhesive tape and positioned so that the foot and the tibia were aligned at approximately 90°. Platinum electrodes were placed subcutaneously in proximity to the tibial nerve to stimulate the plantar flexor muscles. Optimal isometric twitch torque was measured by increasing the current with at least 30-s between each twitch contraction to prevent fatigue. Static performance was assessed through an isometric tetanic contraction at 100 Hz and 125 Hz, each lasting 200-ms, with the ankle positioned at 90° (angle between the tibia and foot). Every isometric contraction was followed by a 2 min rest to prevent neuromuscular fatigue. Next, dynamic eccentric contractions were carried out. The eccentric contractions consisted of a 200-ms isometric contraction immediately followed by a lengthening contraction in which the footplate moved through 30° at a velocity of

40°/s. The plantar flexors were then passively returned to resting length. The susceptibility to contraction-induced force loss was examined through 6 eccentric contractions with 30-s rests between each one. The maximal force of the isometric plateau (occurring during the 200-ms before eccentric stretch) was measured and used to normalize the reduction in force production.

***Ex Vivo* Isolated EDL Muscle Force**

Ex vivo contractile properties of skeletal muscle were evaluated in extensor digitorum longus (EDL) muscles that were surgically removed and analyzed as previously detailed (6, 7). The muscle was quickly immersed in a bath with a physiological salt solution, bubbled with 95% O_2 and 5% CO_2 to keep the pH at 7.4 and maintained at 25°C. The distal tendon was tied to a dual-mode servomotor (Model 1200A Isolated Muscle Test System for Mice; Aurora Scientific, Inc.) and the proximal tendon was clamped to the post directly below the servomotor. After the optimization steps, the maximal isometric tetanic force was measured. Next, the muscle underwent an eccentric contraction protocol that included two eccentric contractions with 3 min rest periods in between. Each eccentric contraction involved an initial 100-ms isometric contraction at optimal frequency immediately followed by stretching the muscle to 30% beyond its optimal length, and then passively returning the muscle to its original length. Following analysis of the muscle contractile properties, the EDL was weighed. The cross-sectional area (CSA) of the EDL was calculated by dividing the EDL mass by the product of fiber length and the density of mammalian skeletal

muscle (1.06 g/cm^3). EDL specific force was calculated by dividing maximal isometric force by the CSA (mN/mm^2).

Antibodies

The following primary antibodies have been described previously and were obtained from the listed sources: affinity-purified β-DG rabbit polyclonal AP83 (4) (Campbell Laboratory); β-DG was also detected by the MANDA G2 7D11 antibody (8) (Campbell Laboratory); matriglycan was detected by the IIH6 monoclonal antibody (9) (Campbell Laboratory; Developmental Studies Hybridoma Bank, University of Iowa; RRID:AB_2617216); recombinant rabbit IIH6 antibody (supplied by Amicus Therapeutics); rabbit polyclonal anti-dystrophin (ab15277; Abcam, San Francisco, CA, USA); anti-beta-sarcoglycan (5B1; Campbell Laboratory); anti-gamma-sarcoglycan (21B5; Campbell Laboratory); polyclonal anti-laminin (L9393; Sigma-Aldrich, St. Louis, MO, USA); monoclonal anti-perlecan (A7L6; Invitrogen, Waltham, MA, USA); anti-neurofilament H (NF-H; CPCA-NF-H; EnCor, Gainesville, FL, USA); anti-synaptophysin (MA5-14532; Thermo Fisher Scientific, Waltham, MA, USA); rabbit anti-utrophin A (abn1739m; Abcam); and monoclonal anti-rapsyn (MA1-746; Thermo Fisher Scientific). Secondary antibodies conjugated to Alexa Fluor 488, 555, 594, and 647 were obtained from Invitrogen.

Histology and Immunofluorescence

Mice were euthanized by cervical dislocation, and skeletal muscle tissues were promptly harvested. The muscle tendons were trimmed, blood was absorbed by wicking, and wet weights were recorded. Muscles were embedded in tissue

freezing medium (Tissue-Tek O.C.T. compound; Sakura FineTek; Torrance, CA, USA) and immediately snap-frozen in liquid nitrogen-cooled 2-methylbutane (isopentane). Ten µm sections were prepared using a cryostat (Leica CM3050S Research Cryostat; Amsterdam, the Netherlands) set at -20ºC. Hematoxylin and eosin (H&E) staining was performed on the cryosections following standard protocols (9). For immunofluorescence, cryosections were blocked with Background Buster (NB306; Innovex Biosciences, Richmond, CA, USA). They were then incubated in primary antibodies overnight, washed with PBS, followed by incubation in secondary antibodies (1:500). After washing with PBS again, the sections were cover-slipped with mounting medium that contained the nuclear marker DAPI (ProLong Gold Antifade Mountant with DAPI; Invitrogen). In some cases, anti-mouse IgG (1:250; Invitrogen) was used to detect muscle fiber damage through immune-cell infiltration and was added together with secondary antibodies. Digital images were captured using either a slide scanner microscope (VS120-S5-FL; Olympus Corporation, Tokyo, Japan) or a confocal laser scanning microscope (FLUOVIEW FV3000; Olympus). Quantitative analysis was conducted using VS-Desktop software (Olympus) and cellSens analysis software (Olympus).

NMJ assessment was conducted as previously detailed (5, 7). EDL, gastrocnemius, and diaphragms were harvested and immediately rinsed three times in PBS for 5 min each. Muscles were then fixed in 4% paraformaldehyde for 20 min followed by three washes in PBS. Fixed EDL and gastrocnemius muscle samples were divided into fiber bundles, while diaphragm muscles

remained intact, before being incubated in 3% Triton X-100/PBS for 3 hr at 4°C. Muscles were then washed in PBS, followed by blocking (Background Buster; Innovex Biosciences) at 4°C for 4 hr. Samples were incubated overnight at 4°C with primary antibodies against NF-H and synaptophysin. The muscles were rinsed in PBS and then incubated for 2 hr with fluorescently conjugated secondary antibodies and Alexa Fluor 488-conjugated α-bungarotoxin (BTX; B13422; Invitrogen). Images were captured with a confocal laser scanning microscope (FLUOVIEW FV3000; Olympus). Complete en face NMJs were identified and captured with Z-stacks using 20x, 60x, and 100x objectives. Maximum intensity Z-stacks were reconstructed with the FV31S software (Olympus) and deconvoluted with cellSens Dimension software (Olympus).

Blinded observers analyzed α-BTX-labeled AChR cluster formations to determine synapse morphology, postsynaptic area, and number of AChR clusters per NMJ. Assessment of synapse morphology involved categorizing synapses based on the presence or absence of irregularities. Postsynapses that displayed the expected pretzel-like morphology were categorized as normal. Abnormal synapse morphology was based on observable irregularities such as extensive fragmentation of AChR clusters, AChR plaques, AChR perforated plaques, and ring-shaped or c-shaped clusters. Categorization of synapse morphology involved evaluation of 80-115 postsynapses per muscle, and the average value for each mouse (n=4 control; n=5 β-DG^{cyto}) was reported. *En face* synapses were captured at 60X or 120X magnification to assess postsynaptic area and fragmentation. Blinded observers used Fiji ImageJ (National Institutes

of Health; Bethesda, MD, USA) to analyze area and fragmentation. The postsynaptic area refers to the total area labeled with α-BTX. AChR clusters per NMJ were determined by the number of identifiable individual α-BTX-labeled fragments within the footprint of the synapse. Twelve to 20 synapses were analyzed from each mouse (n=4 control; n=5 β-DG^{cyto}), and the mean value for each mouse was reported. Determination of innervation status involved evaluating 110-160 NMJs from each mouse (n=4 per group). Analysis was performed on images captured at 40X or 60X magnification. Complete overlap of α-BTX and NF-H/synaptophysin was considered full innervation; incomplete or partial overlap was considered partial innervation; and a postsynapse without a corresponding nerve terminal was considered a non-innervated site. The mean percentages from each mouse were reported. In all cases, NMJs were sampled throughout the muscle tissue.

Statistics

All data in the present study are shown as the means ± standard deviation unless otherwise specified. The number of sampled units, *n*, represents a single mouse in an experiment (i.e., one mouse is *n*=1). All statistical analyses were performed using GraphPad Prism 9 software. A two-tailed t-test was used to compare a single data set with a separate data set. Multiple unpaired t-test with Holm-Sidak post-hoc correction was employed for comparison of innervation status. Differences were deemed statistically significant if the *p*-value was below 0.05.

SI References

1. L. Wan *et al.*, Structure and assembly of the dystrophin glycoprotein complex. *Nature* **637**, 1252-1260 (2025).
2. S. Liu, T. Su, X. Xia, Z. H. Zhou, Native DGC structure rationalizes muscular dystrophy-causing mutations. *Nature* **637**, 1261-1271 (2025).
3. J. S. Satz *et al.*, Visual impairment in the absence of dystroglycan. *J Neurosci* **29**, 13136-13146 (2009).
4. R. A. Williamson *et al.*, Dystroglycan is essential for early embryonic development: disruption of Reichert's membrane in Dag1-null mice. *Hum Mol Genet* **6**, 831-841 (1997).
5. J. M. Hord *et al.*, Sarcolemma resilience and skeletal muscle health require O-mannosylation of dystroglycan. *Skelet Muscle* **15**, 1 (2025).
6. T. Yonekawa *et al.*, Large1 gene transfer in older myd mice with severe muscular dystrophy restores muscle function and greatly improves survival. *Sci Adv* **8**, eabn0379 (2022).
7. H. Okuma *et al.*, N-terminal domain on dystroglycan enables LARGE1 to extend matriglycan on alpha-dystroglycan and prevents muscular dystrophy. *Elife* **12** (2023).
8. A. V. Pereboev, N. Ahmed, N. thi Man, G. E. Morris, Epitopes in the interacting regions of beta-dystroglycan (PPxY motif) and dystrophin (WW domain). *Biochim Biophys Acta* **1527**, 54-60 (2001).
9. J. M. Ervasti, K. Ohlendieck, S. D. Kahl, M. G. Gaver, K. P. Campbell, Deficiency of a glycoprotein component of the dystrophin complex in dystrophic muscle. *Nature* **345**, 315-319 (1990).
